# Chronic Khat Usage as a Cause of Chronic Hypertensive Encephalopathy and Vascular Dementia: A Case Report

**DOI:** 10.7759/cureus.76024

**Published:** 2024-12-19

**Authors:** Jesus R Salas, Rami Ibrahim

**Affiliations:** 1 Neurology, The Ohio State University Wexner Medical Center, Columbus, USA

**Keywords:** chewing khat, dementia, early onset dimentia, khat and health impact, post-stroke dementia

## Abstract

Khat is a native plant of Eastern Africa that is widely utilized for its stimulant-like effects. It is known to have effects similar to those of amphetamine and has a comparable side effect profile, including tachycardia, hypertension, and insomnia. In this case report, we present a 45-year-old Somali gentleman with a history of more than eight years of chronic Khat use, presenting with progressive cognitive decline. MRI findings were significant for white matter changes and microhemorrhages in the setting of chronic and poorly controlled hypertension, likely secondary to chronic Khat use. Understanding, identifying, and educating about the effects of long-term Khat use are important considerations in the treatment and management of hypertension and should be included in the differential diagnosis of vascular dementia and progressive cognitive decline in the Somali population in the United States.

## Introduction

The Khat plant, *Catha edulis*, is native to East Africa and the Arabian Peninsula and has been cultivated since the 7th century. The leaves of the Khat plant contain cathinone, an amphetamine-like alkaloid that produces a psychostimulant effect similar to that of amphetamine and methamphetamine, both of which increase the activity of dopaminergic and noradrenergic transmission [1]. Given the molecular similarities between these compounds, the toxicity associated with long-term use is similar to that seen in chronic amphetamine use, including tachycardia, hypertension, manic behavior, insomnia, increased risk of myocardial infarction, and both acute ischemic and hemorrhagic stroke [2,3]. In this case report, we present a 45-year-old gentleman from Somalia with a reported 10-year history of daily Khat use. He was initially evaluated for acute-on-chronic encephalopathy of unknown etiology and subsequently admitted due to his insidious, stepwise cognitive decline over the past two years, with his family no longer able to care for him. We present a unique case of chronic hypertensive encephalopathy leading to vascular dementia after a long history of poorly controlled hypertension secondary to chronic Khat consumption.

## Case presentation

A 45-year-old right-handed, Somali-speaking gentleman with a past medical history of tobacco use disorder in remission and medication-resistant hypertension on multiple agents, including losartan, hydrochlorothiazide, and nifedipine, initially presented to the emergency department with several weeks of acute-on-chronic confusion, progressive cognitive decline, new-onset aggressive behaviors, and dysarthria. According to family members, the patient had experienced progressive cognitive decline over eight years prior to this presentation and was no longer working.

The patient was accompanied by his daughter, who provided a reliable collateral history. He was initially cooperative during the examination and interview, alert to his name and the year only, but had poor insight and judgment regarding why his daughter brought him to the hospital. His speech was noted to be mildly dysarthric by his daughter, but his fund of knowledge was intact, and he could name objects and repeat phrases appropriately. Family members reported that, over several weeks, the patient exhibited worsening strange behaviors, including wandering through the neighborhood, misplacing kitchen appliances in living rooms and bedrooms, and experiencing intermittent verbal outbursts. They noted that some of these symptoms had occurred in the past, leading to the initiation of Depakote 500 mg daily for behavioral issues two years prior. The family also reported that the patient occasionally appeared to experience complex visual hallucinations involving humanoid figures and animals. The patient often seemed confused and believed he was in Somalia. Social history revealed that he had an extensive social circle and a routine of going to the mosque daily for prayer, followed by socializing. During these gatherings, the patient would smoke or chew Khat leaves for two to three hours at a time. Reportedly, he had engaged in this activity since his early 20s, with increased frequency over the past several years.

Initial vitals on presentation included a blood pressure of 187/104 mmHg, heart rate of 74 beats per minute, and oxygen saturation of 96% on room air. On physical examination, he had no focal neurological deficits apart from a mild left-sided facial droop. Unfortunately, the patient was unable to participate in formal cognitive screening due to poor attention and agitation despite multiple attempts. Given his presentation and the family's inability to care for him, he was admitted to the general medicine team. Reversible biochemical causes of dementia and a stroke evaluation were pursued during admission, as detailed in Table 1. Not listed in the table are results for urine toxicology, urinalysis with reflex to culture, serum autoimmune encephalopathy panel, basic metabolic panel, liver function tests, glucose, magnesium, phosphate, calcium, and complete blood counts, all of which were unremarkable or within normal limits.

**Table 1:** Reversible Biochemical Causes of Dementia and Secondary Stroke Prevention Laboratory Values Listed in the table are laboratory values evaluating for reversible causes of dementia and altered mental status. Additionally, secondary stroke prevention laboratory values, including lipid panel and hemoglobin A1C, were obtained. Not listed in the table are urine toxicology, urinalysis with reflex to culture, serum autoimmune encephalopathy panel, BMP (basic metabolic panel), LFTs (liver function tests), glucose, magnesium, phosphate, calcium, and CBC (complete blood counts) of which were all unremarkable. TSH: thyroid-stimulating hormone, T4: thyroxine, RPR: rapid plasma reagin, HIV: human immunodeficiency virus, HDL: high-density lipoprotein, LDL: low-density lipoprotein.

Laboratory Study	Result	Reference Value
Alcohol	<10 mg/dL	<10 mg/dL
Vitamin B1	101 nmol/L	70-180 nmol/L
Vitamin B12	946 pg/mL	211-911 pg/mL
Folate	114.49 ng/mL	>5.38 ng/mL
TSH	0.968 uIU/mL	0.550-4.780 uIU/mL
T4	1.22 ng/dL	0.89-1.76 ng/dL
Serum RPR	Non-reactive	Non-reactive
HIV-1 / HIV-2 Ab	Non-reactive	Non-reactive
Ammonia	42 umol/L	6-47 umol/L
Serum glucose	133 mg/dL	70-99 mg/dL
Valproic acid - free	5 mcg/mL	5-35 mcg/mL
Valproic acid - total	47 mcg/mL	50-120 mcg/mL
Antinuclear antibody	Negative	Negative
Total cholesterol	202 mg/dL	< 200 mg/dL
Triglycerides	294 mg/dL	<150 mg/dL
HDL	27 mg/dL	>40 mg/dL
LDL	116 mg/dL	< 100 mg/dL
A1C	8.3%	4.7-5.6%

The patient also underwent evaluation for secondary causes of hypertension. A renal ultrasound was performed, which showed no evidence of renal artery stenosis. A transthoracic echocardiogram was conducted and revealed no pathological abnormalities. Serum studies for secondary causes of hypertension were within normal limits, as shown in Table 2.

**Table 2:** Secondary Hypertension Evaluation Laboratory Results Listed above are the laboratory results for the patient's secondary hypertension evaluation. Serum renin, aldosterone, cortisol, metanephrine, and normetanephrine were all found to be within normal limits.

Laboratory Study	Result	Reference Value
Serum renin	33.1 pg/mL	4.2-52.2 pg/mL
Serum aldosterone	32.2 ng/dL	<35.30 ng/dL
Serum cortisol	3.47 mcg/dL	3.09-22.40 mcg/dL
Serum metanephrine	32 pg/mL	<57 pg/mL
Serum normetanephrine	102 pg/mL	<148 pg/mL

**Figure 1:**
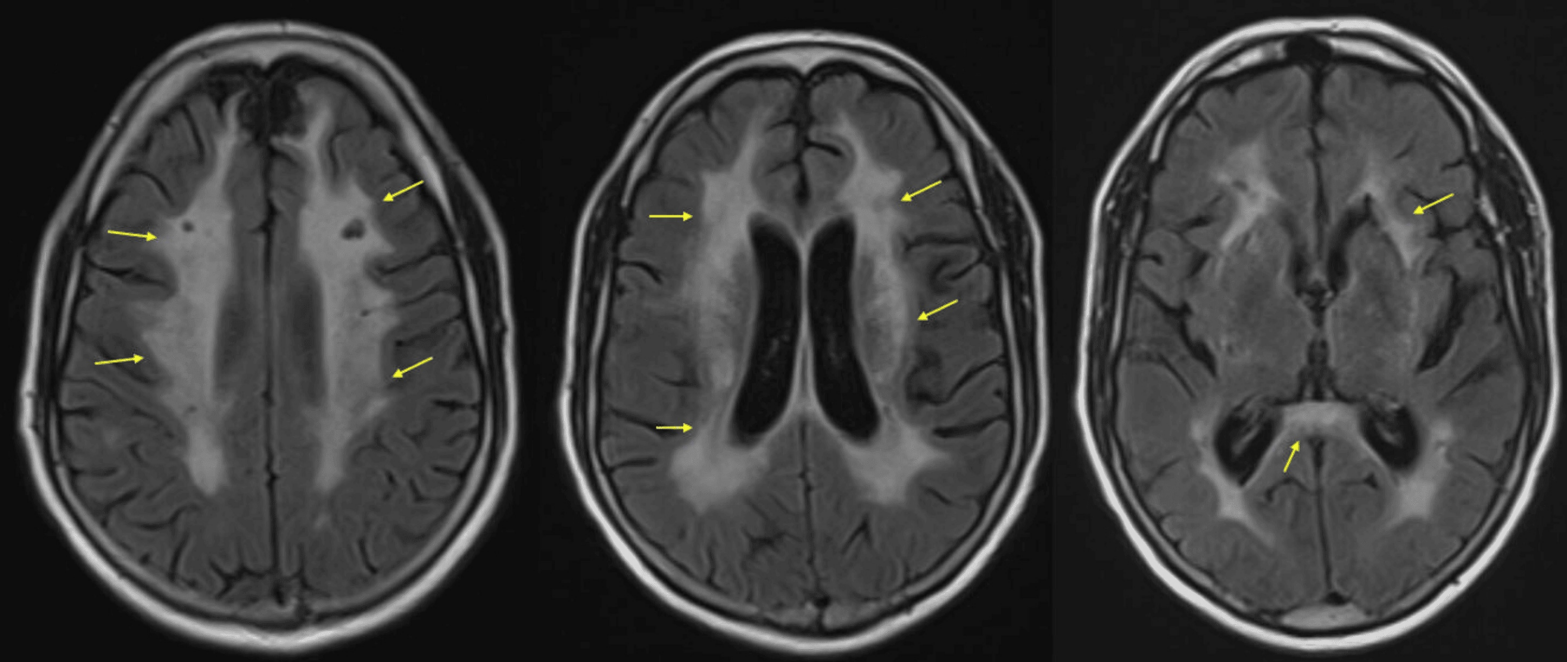
MRI Brain Without Contrast T2 FLAIR Sequence Shown in the image is a series of MRI brain without contrast T2 FLAIR sequence axial images. The panel shows three images with extensive, confluent periventricular white matter T2/FLAIR hyperintensities indicated by the yellow arrows. There are multiple remote lacunar infarcts within the areas of white matter signal abnormalities including bilateral paramedian frontal lobe insults. MRI: magnetic resonance imaging, T2 FLAIR: T2 fluid-attenuated inversion recovery.

**Figure 2:**
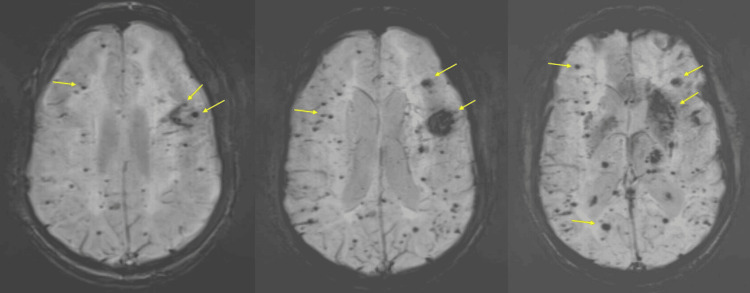
MRI Brain Without Contrast SWI Sequence The image provides a series of axial MRI brain SWI images. Indicated by the yellow arrows are susceptibility hypointensities throughout the bilateral cerebrum representing microhemorrhages. In the panel, there is a prominent juxtacortical area of susceptibility in the left frontal lobe as well as in the left basal ganglia as shown by the yellow arrows. MRI: magnetic resonance imaging, SWI: susceptibility-weighted imaging.

An initial CT of the head revealed stable encephalomalacia in the bilateral medial frontal lobes and extensive confluent low-attenuation cortical white matter. An MRI of the brain, with and without contrast, was performed and is shown in Figures 1, 2. The MRI revealed extensive confluent T2/FLAIR (fluid-attenuated inversion recovery) abnormalities, diffuse microhemorrhages, and multiple cerebral white matter lacunar infarcts. These findings were consistent with severe hypertensive microangiopathy, with possible superimposed white matter changes related to longstanding Khat use. Additional differential considerations included cerebral amyloid angiopathy, toxic encephalopathy, vasculitis, and prior infection or inflammation. A stable juxtacortical left frontal hemorrhage was also noted. No definite areas of restricted diffusion were identified to suggest an acute infarct.

Given that the MRI findings raised concerns for possible CNS vasculitis, our patient underwent additional testing, including a diagnostic cerebral angiogram, which was negative for any acute abnormalities. The patient also underwent a lumbar puncture for vasculitis, dementia, and infectious evaluation, with results detailed in Table 3. He remained hospitalized for six weeks and was discharged to a memory care unit. Due to his autoimmune and vasculitis workup being negative, he was not treated with any immunosuppressive medications or steroids. Ultimately, it was determined that his presentation was secondary to years of uncontrolled blood pressure in the context of chronic Khat use. Risk factor modification, including blood pressure control and cessation of Khat use, was recommended as the best treatment approach. At discharge, he was prescribed atorvastatin 40 mg daily, losartan 50 mg QHS, nifedipine 60 mg XL daily, aspirin 81 mg daily, and metformin 1000 mg daily for stroke risk factor modification. Additionally, he was prescribed Depakote 250 mg BID for mood and agitation. The patient was encouraged to stop using Khat and was referred to addiction medicine for counseling. He had close neurocognitive follow-up in the outpatient setting shortly after discharge.

**Table 3:** Lumbar puncture laboratory results The patient underwent lumbar puncture for evaluation of vasculitis, reversible causes of dementia, and infection. The patient's lumbar puncture and cerebral spinal fluid results were obtained and listed in the table above. The patient had slightly elevated CSF protein and elevated red blood cells present likely secondary to a traumatic spinal tap. Not listed in the table are the meningitis encephalitis panel and CSF VDRL, which were negative. CSF: cerebrospinal fluid, VDRL: venereal disease research laboratory.

Laboratory Study	Result	Reference Value
Glucose	63 mg/dL	40-70 mg/dL
Protein	77 mg/dL	15-45 mg/dL
Total nucleated cells	<3/uL	<6/uL
Red blood cells	7191/uL	<3/uL

## Discussion

In this case report, we present a 45-year-old Somali gentleman with progressive cognitive decline over several years, raising concerns for early-onset dementia. The medical literature has highlighted many effects of Khat use, leading to a variety of medical side effects and complications.

In this specific case, the differential diagnosis was broad. Tuladhar et al. and Van Walleghem et al. reported that patients consuming Khat leaves can experience reversible cerebral vasoconstrictive syndrome, leading to ischemic and hemorrhagic strokes due to Khat's vasoactive properties [4,5]. Based on the initial MRI brain findings, primary CNS vasculitis was considered as part of the differential diagnosis. However, in our case, the lack of recurrent thunderclap headaches in the patient’s history, combined with a negative DCA and reassuring CSF studies, made this diagnosis less likely. Additionally, when reviewing our patient and the MRI findings of several microhemorrhages observed on SWI sequencing, we considered inflammatory cerebral amyloid angiopathy (iCAA). iCAA is commonly a monophasic illness, with patients experiencing mild cognitive impairment for several years, followed by insidious encephalopathy, ultimately leading to rapid cognitive decline over weeks to months. Supporting our final diagnosis, case reports in the medical literature highlight Khat consumption as a potential risk factor for acute cerebrovascular accidents and as a possible mechanism for uncontrolled hypertension. Morrish et al. presented a similar case of a 56-year-old Somali gentleman with psychosis, confusion, and agitation over a period of five weeks [6]. Ultimately, that patient was found to have extensive white matter signal changes on an MRI brain scan, consistent with hypertensive leukoencephalopathy in the context of chronic Khat use over 18 years.

We suspect that Khat's vasoconstrictive properties exacerbated the patient’s poorly controlled stroke risk factors, such as hypertension, hyperlipidemia, and type 2 diabetes. Ultimately, the patient’s prolonged use of Khat over several years led to ischemic and hemorrhagic strokes, culminating in a diagnosis of chronic hypertensive encephalopathy and vascular dementia.

## Conclusions

In conclusion, our case report highlights the importance of obtaining detailed social and substance use histories in patients from Eastern Africa and the Middle East who present with cerebrovascular disorders. For clinical providers, identifying and educating about the effects of long-term Khat use are important considerations in the treatment and management of hypertension. Additionally, Khat use should be included in the differential diagnosis of vascular dementia and progressive cognitive decline in the Somali and East African populations in the United States.

It is also important to acknowledge some limitations of this case report. Confirmatory testing for Khat or Khat-derived metabolites is not commercially available for laboratory testing. In this specific case, the diagnosis of vascular dementia is limited by the absence of documented prior stroke events in the patient’s medical chart or reports from the patient’s family. Furthermore, genetic causes of leukoencephalopathies were not evaluated in this case but should be considered as a possibility. Overall, there are limited case reports in the medical literature highlighting Khat consumption as a potential risk factor for acute cerebrovascular accidents. Physicians across all subspecialties should be aware of Khat as a possible risk factor for cerebrovascular accidents.

## References

[1] Kalix P (1992). Cathinone, a natural amphetamine. Pharmacol Toxicol.

[2] Pehek EA, Schechter MD (1990). Discriminative stimulus properties of (+)cathine, an alkaloid of the khat plant. Pharmacol Biochem Behav.

[3] Bede P, El-Kininy N, O'Hara F, Menon P, Finegan E, Healy D (2017). 'Khatatonia' - cathinone-induced hypertensive encephalopathy. Neth J Med.

[4] Tuladhar AM, Boogaarts HD, de Leeuw FE, van Dijk E (2013). Reversible cerebral vasoconstriction syndrome after chewing khat leaves. Cerebrovasc Dis.

[5] Vanwalleghem IE, Vanwalleghem PW, De Bleecker JL (2006). Khat chewing can cause stroke. Cerebrovasc Dis.

[6] Morrish PK, Nicolaou N, Brakkenberg P, Smith PE (1999). Leukoencephalopathy associated with khat misuse. J Neurol Neurosurg Psychiatry.

